# Multiparameter use of Cardiac Magnetic Resonance as a predictor of pulmonary artery hypertension

**DOI:** 10.1186/1532-429X-18-S1-P148

**Published:** 2016-01-27

**Authors:** Ryan Avery, Rajesh Janardhanan, Elizabeth Krupinski, Ankit Desai

**Affiliations:** 1 grid.134563.6000000012168186XMedical Imaging, University of Arizona, Tucson, AZ USA; 2 grid.189967.80000000419367398Emory University, Atlanta, GA USA; 3 grid.134563.6000000012168186XCardiology, University of Arizona, Tucson, AZ USA

## Background

Cardiac Magnetic Resonance (CMR) provides superior assessment of right ventricle (RV) function and assessment of multiple physiologic parameters with high inter- and intraobserver reproducibility thus providing a noninvasive assessment of pulmonary arterial hypertension (PAH) by evaluating RV physiologic parameters. CMR also provides a unique noninvasive assessment of myocardial fibrosis and quantitative evaluation of RV mass. Our goal was to evaluate the value of CMR to assess RV dysfunction in the setting of PAH using established physiologic parameter in combination with techniques unique to CMR, such as RV mass quantification and T1-mapping.

## Methods

18 patients with PAH (13F, 8M, 40-79 yrs) confirmed by right-heart catheterization underwent CMR (Siemens Aera, 1.5T) with multi-planar steady state free precession (SSFP) sequences. T1-mapping pre- and post-contrast using a modified Look-Locker inversion recovery (MOLLI) sequence and the shortened (ShMOLLI) version were also performed. 13 controls (5F, 8M, 18-76 yrs) were patients referred for CMR to evaluate for ARVD or an echocardiogram abnormality and were normal on CMR. Post-processing software was performed to determine biventricular ejection fractions (EF) and myocardial mass. Pulmonary and tricuspid annular plane systolic excursion (PAPSE & TAPSE) and MPA size were measured in RVOT, 4-chamber, and LVOT views respectively. Left ventricle eccentricity index in end diastole (LVEId) and end systole (LVEIs) were performed in the in a mid short-axis slice. Parametric T1 values were identified by ROI placement on the interventricular septum and LV blood pool before and after contrast to generate a partition coefficient. A logistic regression comparing PAH patients to controls as the dependent variable was performed. RVEF, RV mass, PAPSE, TAPSE, LVEId, LVEIS, and MPA size were used as independent variables. For T1-mapping, the T1 septal values before and after contrast, and partition coefficients were evaluated as independent variables.

## Results

RVEF, RV mass, PAPSE, and MPA size were significant predictors of PAH. RVEF (mean 34.86 ± 12.11, p = 0.043) and MPA size (mean 3.31 ± 0.55, p = 0.006) are well established PAH predictors. PAPSE in PAH patients (mean 8.98 ± 4.56, p = 0.026) was lower compared to controls (mean 13.20 ± 2.74), and met statistical significance compared to TAPSE (p=0.319). Increased RV mass (mean 39.86 ± 13.01, p = 0.010) in PAH patients was found compared to controls (mean 23.70 ± 6.20). T1-mapping showed that only pre-contrast ShMOLLI T1 signal measurements correlated with PAH (p=0.007). Multiple regression confirmed that the most significant imaging findings were MPA size, RV mass, and PAPSE respectively.

## Conclusions

CMR offers unique quantitative assessment of RV mass and PAPSE, which are significant predictors of PAH. Given current use of CMR for RVEF calculation for PAH prognosis, CMR with the addition of RV mass and PAPSE should be considered for first-line noninvasive PAH diagnosis.

